# Pictorial key for females of *Decevania* Huben (Hymenoptera, Evaniidae) and description of a new species

**DOI:** 10.3897/zookeys.116.1473

**Published:** 2011-07-07

**Authors:** Ricardo Kawada

**Affiliations:** Museu de Zoologia da Universidade de São Paulo. Av. Nazaré, 481, Ipiranga, CEP 04263-000. São Paulo-SP, Brazil

**Keywords:** Evanioidea, taxonomy, new species

## Abstract

*Decevania* Huben currently comprises 13 species, the females of which are known for only four. Herein an additional Neotropical *Decevania* is newly described: *Decevania feitosai* Kawada, **sp. n.** from Colombia. The description and identification key were made using the DELTA program. A pictorial key to females of *Decevania* is provided. Anatomical terminology follows the Hymenoptera Anatomy Ontology project with an atlas for terminologies used for recognition of *Decevania* species. The distribution maps can be accessed in Google Maps or through of Dryad (repository of data).

## Introduction

*Decevania* Huben1 is a small genus of Neotropical Evaniidae with 13 species recognized so far. Kawada and Azevedo (2007) recently revised the genus, providing redescriptions of *Decevania parva*2 (Enderlein, 1901) and *Decevania striatigena*3 (Kieffer, 1910), descriptions of 11 new species, an identification key, illustrations of all species, and increased the geographical distribution known for the genus, which ranges from Mexico to Bolivia, east to Brazil.

Species in this genus are characterized by having 8 flagellomeres, relatively reduced eyes (usually females), wings frequently large and floppy with reduced venation (C, Sc, M+CU, 1CUa, 1CUb and 2CU only present), fore wing with only one cell enclosed by tubular veins (costal), and hind tarsomeres 1-3 elongated posteriorly into spines. According to Kawada and Azevedo (2007), *Decevania* resembles *Hyptia* Illiger4 by having one closed cell in the fore wing always with M+CU, 1CU, and 2CU veins combined (a Caribbean group of *Hyptia* has a close configuration). However, *Decevania* has the stigmal vein wide (narrow in *Hyptia*), 1R1 vein shorter (longer in *Hyptia*) and body with sparse punctures (usually dense punctures in *Hyptia*).

*Decevania* species are sexually dimorphic (antenna, eye, color, facial sculpture, and others) and this complicates association of the sexes and description of new taxa. The head in females is distinctly sculptured and eyes flattened. The antenna is enlarged progressively from the fourth flagellomere apically, antennal pubescence is considerably reduced in flagellomeres IV–X (flattened area) and the posterior region of the metasoma is expanded dorso-ventrally with the ovipositor usually concealed. Males generally have a larger bulging eye, all flagellomeres are equal in diameter, antennal pubescence is evenly distributed with long setae interspersed and the posterior region of the metasoma is constricted dorsoventrally with genitalia protracted, depending on preservation.

The goal of this paper is to disseminate the pictorial key for females of *Decevania* and describe a new species of this genus from Colombia.

## Material and methods

**Material.** The material examined is presented in a list of museums with respective acronyms and countries: CNCI (Canadian National Collection of Insects5) and IAVH (Instituto de Investigación de Recursos Biológicos Alexander von Humboldt, Colombia6). The holotypes are unambiguously identifiable by mean of a red holotype label. The type-material of newly described species are deposited in the IAVH and MZSP (Museu de Zoologia da Universidade de São Paulo, Brazil7).

**Images.** The best characters for distinguishing species were photographed under a stereomicroscope Leica M205C, magnifying glass attached to video camera Leica DFC 295. The equipment responsible for storing and processing data was a desktop computer with Windows 7 Professional and high-capacity processor Intel (R) Xeon (R) CPU and the software used to combine the images was Leica LAS (Leica Application Suite V3.6.0) Microsystems by Leica8 (Switzerland) Limited. Photos were edited in PhotoShop® using the adjustments (e.g., levels, shadows/highlights), tools (e.g., healing brush, clone stamp) and filters (e.g., unsharp mask).

**Distribution map.** Google maps9 provides a powerful tool for fast, collaborative research, with some advantages listed below: (1) steady inclusion of data even after publication; (2) use of the same map in other publications, enabling a comparison with previous work; (3) fast inclusion of data through a network of collaboration; (4) accuracy and standardization of data among researchers; (5) the use of the same map, resulting from a publication, elsewhere in the network (blog’s, discussion list, meetings). The locality data reported for all analyzed specimens are a literal transcription of the label. Details on the data associated with these specimens may be accessed at the following link, using Google url shortener10: Decevania Distribution11 in © 2011 Google - Map data © Google or downloaded through the Dryad12, an international repository of data underlying peer-reviewed articles.

**Taxonomic procedures.** The taxonomic treatment method follows Winston (1999). The description and identification key were made using the DELTA program. Morphological characters for species of *Decevania* were imported to the DELTA editor (Description Language for Taxonomy13) (Dallwitz 1980, Dallwitz et al. 1999). The species description was generated by DELTA <tonart> with output in the format of “character: character state(s)”; Identification key by DELTA <key> (Dallwitz 1980, Dallwitz et al. 1993). The dichotomous, pictoral identification key follows the procedures of Winston (1999). For the purpose of this description, the new species are diagnosable by putative autapomorphies or by a unique combination of fixed character states.

**General terminology.** Anatomical terminology follows the Hymenoptera Anatomy Ontology project (HAO14) using the proofing tool available through the Hymenoptera Glossary 15 (Yoder et al. 2010). Some terms are also included from Deans and Huben (2003) and Kawada and Azevedo (2007). The list of terminology is illustrated and labeled to facilitate their use (see table 1).

## Pictorial key for females of Decevania

(Unknown female for *Decevania brevis* Kawada, 2007; *Decevania deansi* Kawada, 2007; *Decevania destituta* Kawada, 2007; *Decevania elongata* Kawada, 2007; *Decevania glabra* Kawada, 2007; *Decevania hemisphaerica* Kawada, 2007; *Decevania nigra* Kawada, 2007; *Decevania polita* Kawada, 2007; *Decevania striatigena* (Kieffer, 1910))

Fig. a1–25

**Figures a1–6.**
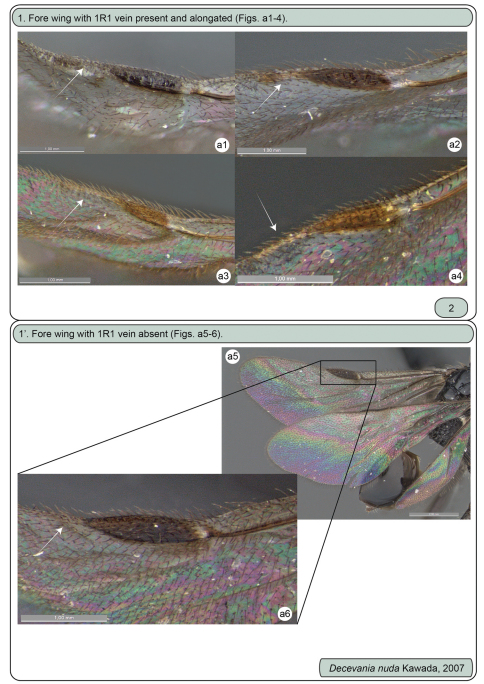
Females. **a1** *Decevania feitosai* sp. n., left fore wing: 1R1 vein **a2** *Decevania parva* (Enderlein, 1901), left fore wing: 1R1 vein **a3** *Decevania reticulata* Kawada, 2007, left fore wing: 1R1 vein **a4** *Decevania unidentata* Kawada, 2007, holotype, left fore wing: 1R1 vein **a5-6** *Decevania nuda* Kawada, 2007 **a5** wings and **a6** left fore wing: 1R1 vein.

**Figures a7–10.**
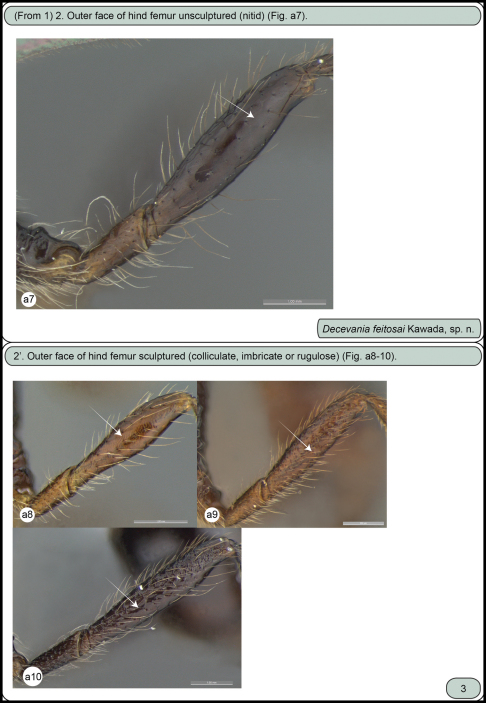
Females. **a7** *Decevania feitosai* sp. n., left hind femur in lateral view **a8** *Decevania unidentata* Kawada, 2007, holotype, left hind femur in lateral view **a9** *Decevania reticulata* Kawada, 2007, left hind femur in lateral view **a10** *Decevania parva* Kawada, 2007, left hind femur in lateral view.

**Figures a11–19.**
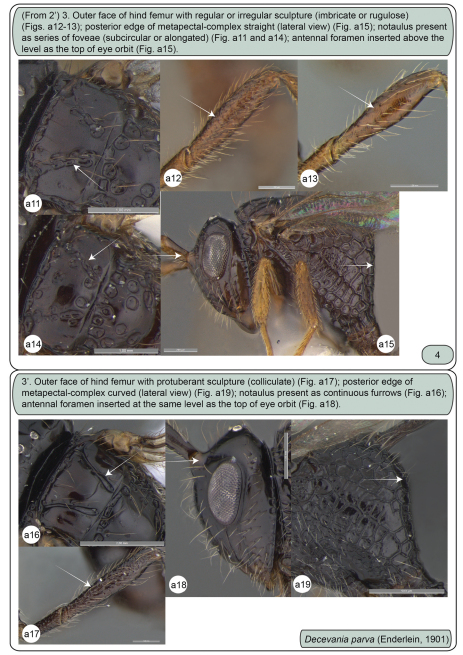
Females. **a11** *Decevania reticulata* Kawada, 2007, mesoscutum in dorsal view **a12** *Decevania reticulata* Kawada, 2007, left hind femur in lateral view **a13** *Decevania unidentata* Kawada, 2007, holotype, left hind femur in lateral view **a14** *Decevania unidentata* Kawada, 2007, holotype, mesoscutum in dorsal view **a15** *Decevania reticulata* Kawada, 2007, head and mesosoma in lateral view **a16–19** *Decevania parva* (Enderlein, 1901) **a16** mesoscutum in dorsal view **a17** left hind femur in lateral view **a18** head in lateral view and **a19** metapectal-propodeal complex in lateral view.

**Figures a20–25.**
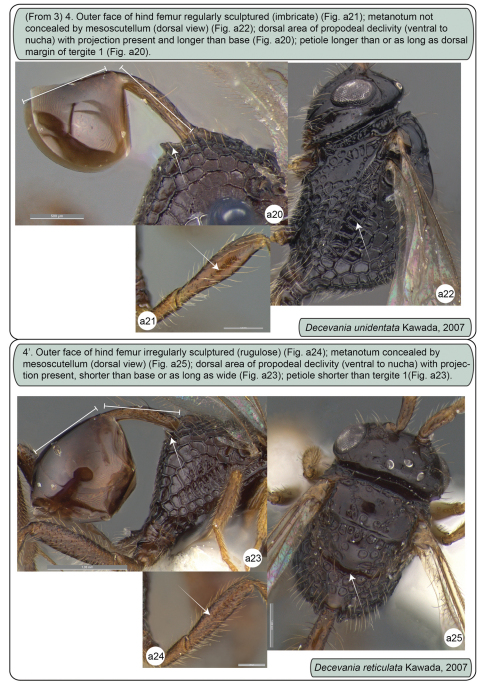
Females. **a20-22** *Decevania unidentata* Kawada, 2007, holotype **a20** propodeum and metasoma in lateral view **a21** left hind femur in lateral view **a22** head and mesosoma in dorsal view **a23–25** *Decevania reticulata* Kawada, 2007 **a23** propodeum and metasoma in lateral view **a24** left hind femur in lateral view and **a25** head and mesosoma in dorsal view.

### Decevania feitosai

Kawada sp. n.

urn:lsid:zoobank.org:act:C11F980C-6446-4311-B71B-6812939BEC49

http://species-id.net/wiki/Decevania_feitosai

**Figure 1.**
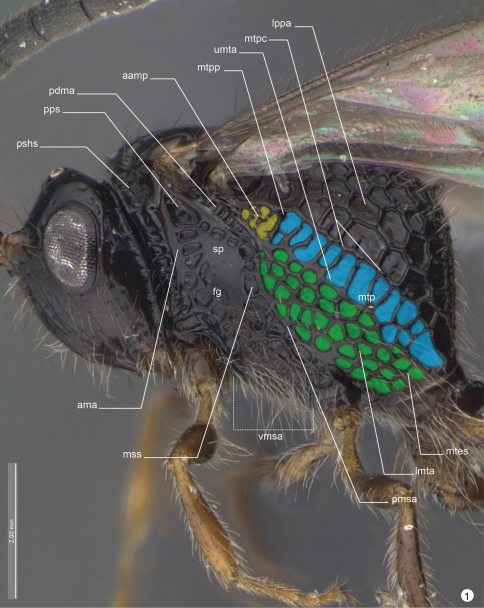
*Decevania feitosai* sp. n. Holotype, female. Head and mesosoma in lateral view. For terminology see the list in Material and methods. Scale in the figure.

Fig. 1 [Fig F6] [Fig F7] [Fig F8] [Fig F9] [Fig F10] 12 

#### Description.

Female body length: 1.6 mm (head to propodeum). Head color: black. Mesosoma color: black. Legs color: fore leg: trochanter, trochantellus, tibia, tarsus light-castaneous; femur dark-castaneous. Wings: fore and hind wing hyaline. Metasoma: petiole light-castaneous; tergites: dark-castaneous.

**Figure 2. F6:**
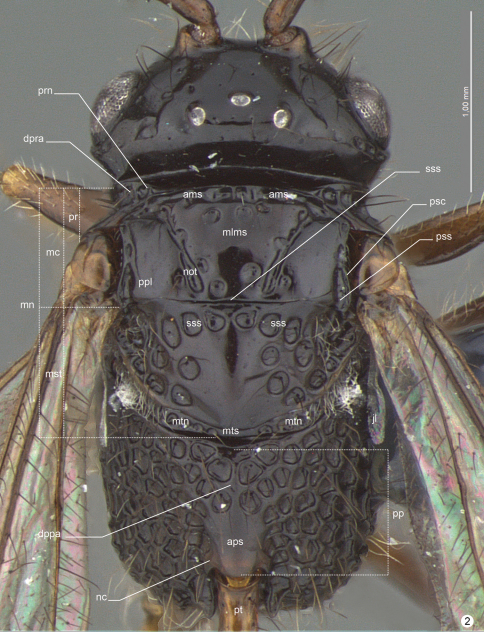
*Decevania feitosai* sp. n. Holotype, female. Head and mesosoma in dorsal view. For terminology see the list in Material and methods. Scale in the figure.

*Head* (Fig. 1-2, 5-7, 9). Head: long and stiff setae present evenly distributed; close to mesosoma. Vertex: slightly convex in lateral view; nitid with some small and sparse punctures. Ocelli: equal in size; arranged in obtuse isosceles triangle; anterior ocellus: separated from posterior ocellus by one ocellar diameter; anterior ocellus: not reaching the imaginary line between the anterior margin of posterior ocelli; posterior ocelli: separated by three ocellar diameters. Upper face: nitid with some sparse punctures. Eye: subovoid (lateral view); detached from dorsal profile of head; height of eye: as high as anterior margin of mesopleuron. Circumocular sulcus: absent. Postorbital carina: present; extending from anterior base of mandible to 3/4 the height of eye; strongly sinuous; narrower than postgenal sulcus. Antennal foramen: positioned at the same level as the top of the eye orbit; separated by one antennal foramen diameter; antennal rim: elevated laterally. Scape: long and stiff setae present evenly distributed; as long as F8. Pedicel + flagellomere 1: longer than wide; pedicel: as long as F1; flagellum: evenly and densely setose with some sparse and long setae. Median process of lower face: very weak in lateral view (difficult to see). Orbital band: strong, narrow and straight striae to the ventral margin of antennal foramen. Malar sulcus: present and conspicuous, differs from orbital band striae. Malar space: 0.64 times the height of eye (greater length). Clypeus: projecting medially; apical margin dilated and convex laterally. Mandible: two visible teeth, apical tooth longer and sharper than basal tooth.

*Mesosoma* (Fig. 1-2, 9, 11-12). Pronotum: long and stiff setae present evenly distributed. Pronotal neck: obscured. Dorsal pronotal area: concealed medially. Dorsolateral area of pronotum: expanded posteriorly into a lobe. Pronotal suprahumeral sulcus: scrobiculate, with a large fovea anterior to the lobe. Transverse pronotal carina: acuminate and extending along the anterior margin of pronotum. Mesothoracic spiracular incision: strongly curved and almost closed into an orifice. Lateral and dorsolateral pronotal area: not clearly separated by a carina (inconspicuous). Lateral pronotal area: narrow, same width between the upper eye orbit and occipital carina (widest point); vertical and covered by a row of fovea (transverse pronotal sulcus). Mesonotum: slightly raised (lateral view: compared with propodeum). Mesoscutum: 2.0 times wider than long; nitid with a few, sparse and regular foveae. Anterior mesoscutal sulcus: present as continuous furrow. Notaulus: present as continuous furrow, slightly curved towards the middle and not reaching the posterior margin. Median lobe of mesoscutum: slightly curved anteriorly (lateral view: difficult to see). Parascutal carina: present at posterior half; sulcus: following the parascutal carina and opening posteriorly. Parapsidal line: conspicuous suture, same length of parascutal carina and reaching the posterior margin of mesoscutum. Transscutal articulation: open in the middle and closing to lateral, near the parapsidal line. Mesoscutellum: long and stiff setae present, evenly distributed laterally; nitid in the middle with closed fovea laterally; bulging posteromedially; with a delicate median convexity on the posterior margin, but without overlap on metanotum. Scutoscutellar sulcus: not reaching the transscutal articulation, covered by a large and subcircular fovea. Metanotum: dorsolateral area covered by moderate (cuticle visible) layer of setae. Metanotum and metascutellum: form a continuous structure. Metascutellum: as a flat and nitid structure. Epicnemial carina: without median process (continuous shape). Prespecular sulcus: composed of one fovea. Anterior mesopleural area: covered by a row of rectangular impressions to femoral groove. Speculum: slightly dilated just above the middle of femoral groove. Mesepimeral sulcus: present as a row of irregular and subcircular foveae from posterodorsal mesepimeral area to mesocoxal foramen. Posterodorsal mesepimeral area: scrobiculate (narrow and shallow). Posterior mesepimeral area: curve and elongated posteriorly (closer to metacoxal foramen). Femoral groove: weakly concave; unsculptured medially. Mesopleural pit: absent. Ventral mesopleural area: covered by a subcircular and adjacent fovea; long and stiff setae present evenly distributed. Mesosternum: higher compared to metasternum; mesosternum foveate (irregular) with an open area (punctate) laterally. Mesodiscrimen: present as a flat and inconspicuous sulcus. Mesocoxa: distant 2.5 times (width of mesocoxa) from procoxa; adjacent to metacoxa. Meso- and metacoxa: without a pair of processes between coxae. Metapleuron (metapleural arm to metacoxal foramen): at least 3 times longer than wide. Metapleural carina: straight and parallel with concave lower metapleural area. Upper metapleural area: covered by a row of rectangular foveae. Lower metapleural area: lower region covered by an irregular polygonal fovea; long and stiff setae present, evenly distributed. Metapleural pit: present. Anterior area of metapleural pit: acute isosceles triangle shaped and covered by an irregular fovea. Metapleural epicoxal sulcus: present as a row of large and subrectangular foveae. Metanotum and propodeum: form a continuous structure. Propodeum: irregular foveae (dorsal) to regularly areolate (lateral). Dorsal propodeal area: long and stiff setae present, evenly distributed. Lateral propodeal carina: absent. Lateral propodeal area (upper region): long and stiff setae present evenly distributed. Adpetiolar strip: longer than wide. Nucha: slightly elevated (lateral view). Upper region of propodeal declivity (ventral to nucha): projection present and longer than base. Middle area of propodeal declivity: with long and stiff setae present evenly distributed. Posterior edge of metapectal-complex: curved (lateral view).

*Legs* (Fig. 4, 10). Protibial spur: apex of calcar longer than apex of velum. Hind leg: long and stiff setae present evenly distributed (longer than outer spur); nitid with sparse punctures (trochanter, trochantellus, femur and tibia). Trochanter: 3.6 times longer (longer point) than wide (widest point). Hind femur: dorsal and ventral margin slightly dilated medially. Hind tibia: longer than hind femur; apical incision of hind tibia: sinuous. Tibial spurs: slightly sinuous; inner tibial spur: extending past the mid length of basitarsus; outer tibial spur: 1.8 times the length of hind basitarsus. Tarsus: minute striae (interspace) and more closer punctures; projections: conspicuous in tarsus 1–3; basitarsus: as long as tarsus 2–4 combined; basitarsus projection: longer than apex of basitarsus (widest point). Tarsal claw: hook-shaped, medially with a minute ventral spine.

*Wings* (Fig. 8). Apex of fore wing: bordered by long setae. Costal cell: the same length as head + mesosoma combined (dorsal view). Stigmal vein: as wide as costal cell. 1R1 vein: as long as stigmal vein, with slightly dilated apex. M+CU, 1CU and 2CU veins combined: extending past the propodeal declivity. 1CUb and 2CU vein: combined to form an angulated angle (45 degrees). 2CU vein: present with a slight dilatation distally. Hind wing: three hook-shaped hamuli of equal size; fusiform and three times longer than wide. Jugal lobe: present, slender and extending past the propodeal spiracle.

*Metasoma* (Fig. 11). Petiole: shorter than propodeal declivity; 6–7 times longer than wide; slightly curved distally. Transverse carina on petiole: as a narrow and acuminate rim. Dorsal petiolar area: nitid. Lateral petiolar area: some sparse and elongated punctures; long and stiff setae present, evenly distributed. Ventral petiolar area: fine and delicate longitudinal carina. Metasoma: subovoid (lateral view) with ovipositor concealed; without setae except T6–7 on posterior edge. Tergite 1: longer than petiole.

**Figures 3–5. F7:**
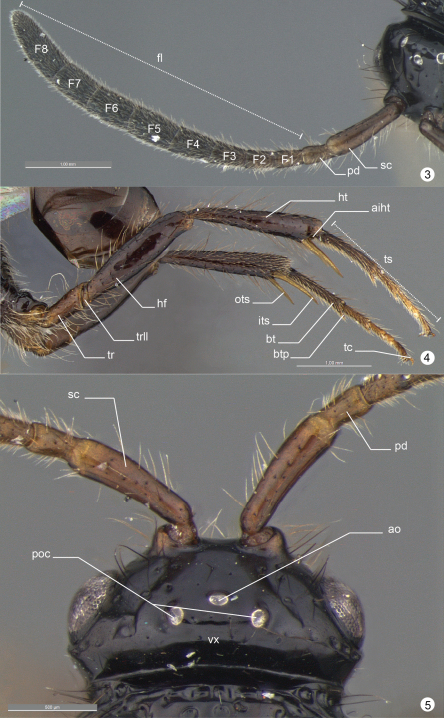
*Decevania feitosai* sp. n. Holotype, female. **3** right antenna in dorsal view **4** hind legs in lateral view **5** head in dorsal view. For terminology see the list in Material and methods. Scale in the figures.

**Figures 6–7. F8:**
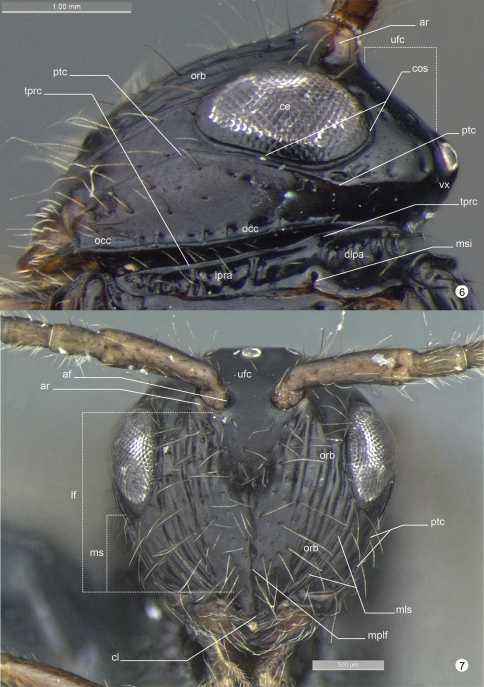
*Decevania feitosai* sp. n. Holotype, female. **6** head in lateral view **7** head in frontal view. For terminology see the list in Material and methods. Scale in the figures.

**Figures 8–9. F9:**
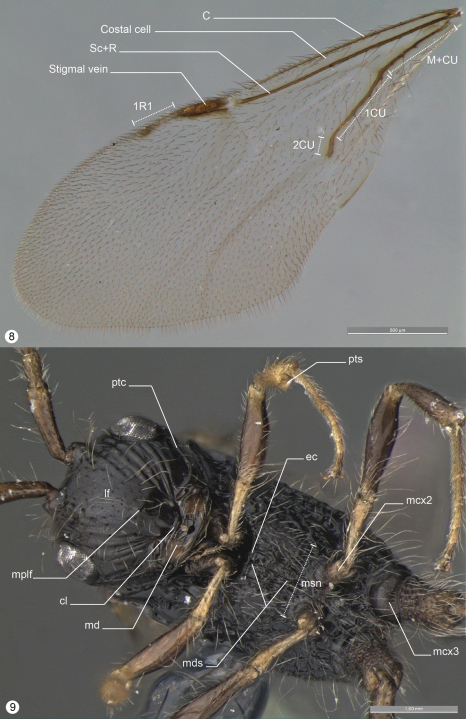
*Decevania feitosai* sp. n. Holotype, female. **8** left fore wing **9** head and mesosoma in ventral view. For terminology see the list in Material and methods. Scale in the figures.

**Figures 10–11. F10:**
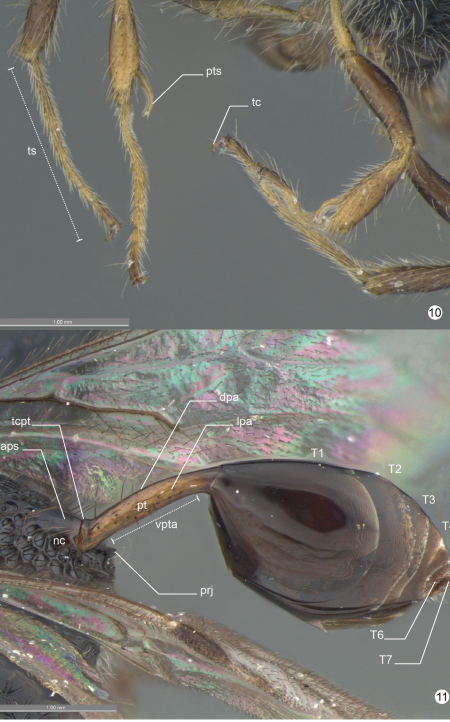
*Decevania feitosai* sp. n. Holotype, female. **10** fore and mid leg in frontal view **11** metasoma in laterodorsal view. For terminology see the list in Material and methods. Scale in the figures.

**Figure 12.**
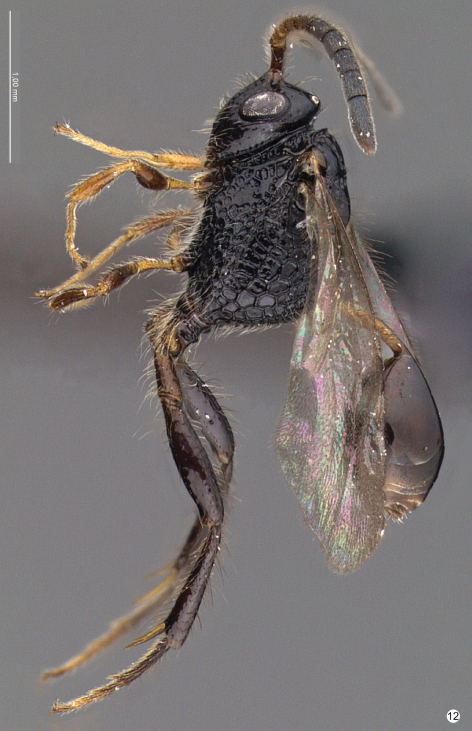
*Decevania feitosai* sp. n. Holotype, female. **10** habitus in lateral view. Scale in the figure.

#### Diagnosis.

Eye: 1.8–2.0 times higher than wide. Postorbital carina: present and complete; conspicuously outlined; detached from the margin of lower eye orbit; sinuous (see malar space); reaching the top of eye orbit (some foveae may also be present and are part of carina). Antennal foramen: inserted at the same level as the top of eye orbit; antennal rim: conspicuously elevated laterally (head lateral view). Median lobe of mesoscutum: slightly curved or flat (lateral view). Notaulus: present as continuous furrow. Metanotum: not concealed by mesoscutellum (dorsal view). Sculpture of hind femur: unsculptured (nitid, autapomorphy for *Decevania feitosai* sp. n.). Posterior edge of metapectal complex: curved (lateral view). Dorsal area of propodeal declivity (ventral to nucha): projection present and longer than base. Petiole: longer than or as long as dorsal margin of tergite 1. 1R1 vein: present and elongate.

#### Etymology.

The specific epithet is a patronymic honoring Rodrigo M. Feitosa, colleague and researcher of Formicidae from MZSP.

#### Link to distribution map.

Decevania Distribution16.

#### Material examined.

Holotype. Female. **COLOMBIA:** Risaralda, SFF Otún Quimbaya, El Molinillo, 4°43'N, 75°34'W , 2220 m, Malaise, 17.ii–04.iii.2003, G. López leg., M.3696 (IAVH). Paratypes. 3 females. **COLOMBIA:** Magdalena, PNN Sierra Nevada de Santa Marta, San Lorenzo, 10°48'N, 73°39'W , 2200 m, Malaise, 09–24.vi.2000, J. Cantillo leg. M. 205 (IAVH 65814); 09–24.vi.2000, J. Cantillo leg. M. 205 (IAVH 65815); 24–30.vi.2000, J. Cantillo leg. M. 211 (IAVH 65816). 4 females. Risaralda, SFF Otún Quimbaya, El Molinillo, 4°43'N, 75°34'W , 2220 m, 03–17.xii.2002, Malaise, G. Walker leg., M. 2972 (IAVH 65827); 4°43'N, 75°34'W , 2220 m, 17.xii.2002–03.i.2003, Malaise, G. Walker leg., M. 2971 (IAVH 65828). Cuchilla Camino, 4°43'N, 75°35'W , 2050 m, 04–17.ii.2003, Malaise, G. López leg., M. 3680 (IAVH). Cuchilla Camino, 4°44'N, 75°35'W , 1960 m, 04–21.iii.2003, Malaise, G. López leg., M. 3669 (IAVH 65826).

## Additional diagnoses for females of Decevania

*Decevania nuda* Kawada, 2007. Eye: 1.8–2.0 times higher than wide. Postorbital carina: present and complete; conspicuously outlined; closer to the margin of lower eye orbit; slightly sinuous (see malar space); reaching the top of eye orbit (some foveae may also be present and are part of carina). Antennal foramen: positioned above the level of the top of eye orbit; antennal rim: inconspicuous elevated laterally (head lateral view). Median lobe of mesoscutum: curved (lateral view). Notaulus: present as series of elongate foveae. Metanotum: not concealed by mesoscutellum (dorsal view). Sculpture of hind femur: protuberant sculpture (colliculate). Posterior edge of metapectal complex: angulated (lateral view). Dorsal area of propodeal declivity (ventral to nucha): projection present, shorter than base or as long as wide. Petiole: shorter than tergite 1. 1R1 vein: absent.

### Material examined.

Paratype. Female. **ECUADOR:** Napo, Sierra Azul, 0.67°S, 77.92°W , 2300 m, 21–22.iv.1996, PT, P.J. Hibbs col. (CNCI).

*Decevania parva* (Enderlein, 1901). Eye: 1.8–2.0 times higher than wide. Postorbital carina: present and complete; inconspicuously outlined; detached from the margin of lower eye orbit; sinuous (see malar space); not reaching the top of eye orbit. Antennal foramen: positioned at the same level as the top of eye orbit; antennal rim: conspicuous elevated laterally (head lateral view). Median lobe of mesoscutum: curved (lateral view). Notaulus: present as continuous furrow. Metanotum: not concealed by mesoscutellum (dorsal view). Sculpture of hind femur: protuberant sculpture (colliculate). Posterior edge of metapectal complex: curved (lateral view). Dorsal area of propodeal declivity (ventral to nucha): projection present, shorter than base or as long as wide. Petiole: longer than or as long as dorsal margin of tergite 1. 1R1 vein: present and elongated.

### Material examined.

Female. **COLOMBIA:** Cundinamarca, PNN Chingaza Bosque, Palacio, 4°31'N, 73°45'W , 2930 m, Malaise, 20.xii.2000–05.i.2001, L. Cifuentes leg., M. 1223 (IAVH 65781).

*Decevania reticulata* Kawada, 2007. Eye: 1.8–2.0 times higher than wide. Postorbital carina: present and complete; conspicuously outlined; detached from the margin of lower eye orbit; slightly sinuous (see malar space); reaching the top of eye orbit (some foveae may also be present and are part of carina). Antennal foramen: positioned above the level as the top of eye orbit; antennal rim: inconspicuously elevated laterally (head lateral view). Median lobe of mesoscutum: curved (lateral view). Notaulus: present as series of subcircular foveae. Metanotum: concealed by mesoscutellum (dorsal view). Sculpture of hind femur: irregular sculpture (rugulose). Posterior edge of metapectal complex: angulated (lateral view). Dorsal area of propodeal declivity (ventral to nucha): projection present, shorter than base or as long as wide. Petiole: shorter than tergite 1. 1R1 vein: present and elongated.

### Material examined.

Paratype. Female. **COLOMBIA:** Chocó, PNN Utría Cocalito Dosel, 6°1'N, 77°20W , 20 m, Malaise, 04–19.vii.2000, J. Pérez leg., M. 339 (IAVH 65778).

*Decevania unidentata* Kawada, 2007. Eye: 1.6 times higher than wide. Postorbital carina: present, but some portion not visible; inconspicuously outlined; closer to the margin of lower eye orbit; reaches the top of eye orbit (some foveae may also be present and are part of carina). Antennal foramen: positioned above the level as the top of eye orbit; antennal rim: conspicuous elevated laterally (head lateral view). Median lobe of mesoscutum: slightly curved or flat (lateral view). Notaulus: present as series of subcircular foveae. Metanotum: not concealed by mesoscutellum (dorsal view). Sculpture of hind femur: regular sculpture (imbricate). Posterior edge of metapectal complex: angulated (lateral view). Dorsal area of propodeal declivity (ventral to nucha): projection present and longer than base. Petiole: longer than or as long as dorsal margin of tergite 1. 1R1 vein: present and elongate.

**Material examined.** Holotype observed. Access through: Evanioidea online17.

## Supplementary Material

XML Treatment for Decevania feitosai

## Appendix I

**Table:** **Abbreviations used on figures**

abbreviation	description	detail	Fig.
aamp	anterior area of metapleural pit	area anterior to metapleural pit, after posterodorsal mesepimeral area and bellow the propodeal spiracle	1
af	antennal foramen		7
ar	antennal rim		6, 7
aiht	apical incision of hind tibia	distal margin of hind tibia, site of insertion of inner and outer tibia spurs	4
ama	anterior mesopleural area		1
ams	anterior mesoscutal sulcus		2
ao	anterior ocellus		5
aps	adpetiolar strip		5
bt	basitarsus		4
btp	basitarsus projection	projection of the apex of tarsus (at least tarsus 1-3)	4
ce	compound eye		6
cl	clypeus		7, 9
cos	circumocular sulcus	furrow that surrounds the compound eye	6
dlpa	dorsolateral pronotal area		6
dpa	dorsal petiolar area		11
dppa	dorsal propodeal area		2
dpra	dorsal pronotal area		2
ec	epicnemial carina		9
fg	femoral groove		1
fl	flagellum		3
F1, F2...	flagellomere		3
hf	hind femur		4
ht	hind tibia		4
its	inner tibial spur		4
jl	jugal lobe		2
lf	lower face		7, 9
lmta	lower metapleural area		1
lpa	lateral petiolar area		11
lppa	lateral propodeal area		1
lpra	lateral pronotal area		6
mc	mesoscutum		2
mcx2	mesocoxa		9
mcx3	metacoxa		9
md	mandible		9
mds	mesodiscrimen		9
mlms	median lobe of mesoscutum		2
mls	malar sulcus		7
mn	mesonotum		2
mplf	median process of lower face		7, 9
ms	malar space		7
msi	mesothoracic spiracular incision		6
msn	mesosternum		9
mss	mesepimeral sulcus		1
mst	mesoscutellum		2
mtes	metapleural epicoxal sulcus		1
mtn	metanotum		2
mtp	metapleuron	divided into two regions by difference of sculpture and flatness: upper metapleural area (usually flat/areolate) and lower metapleural area (usually concave/foveolate)	1
mtpc	metapleural carina		1
mtpp	metapleural pit		1
mts	metascutellum		2
nc	nucha		2, 11
not	notaulus		2
orb	orbital band		6, 7
ots	outer tibial spur		4
pd	pedicel		3, 5
pdma	posterodorsal mesepimeral area		1
occ	occipital carina		6
pmsa	posterior mesepimeral area		1
poc	posterior ocelli		5
pp	propodeum		2
ppl	parapsidal line		2
pps	prespecular sulcus		1
pr	pronotum		2
prj	projection of propodeum		11
prn	pronotal neck		2
psc	parascutal carina		2
pshs	pronotal suprahumeral sulcus		1
pss	parascutal sulcus		2
pt	petiole		11
ptc	postorbital carina		6, 7
pts	protibial spur		10
sc	scape		3, 5
sp	speculum		1
sss	scutoscutellar sulcus		2
T1, T2...	tergite		11
tc	tarsal claw		4, 10
tcpt	transverse carina on petiole		11
tprc	transverse pronotal carina		6
tr	trochanter		4
trll	trochantellus		4
ts	tarsus		4, 10
tsa	transcutal articulation		2
ufc	upper face		6, 7
umta	upper metapleural area		1
vmsa	ventral mesopleural area		1
vpta	ventral petiolar area		11
vx	vertex		5, 6

## Appendix II

**Table:** **Links**

1	http://evanioidea.info/public/taxon_name/show/25686	introduction
2	http://evanioidea.info/public/taxon_name/show/25752	introduction
3	http://evanioidea.info/public/taxon_name/show/25753	introduction
4	http://evanioidea.info/public/taxon_name/show/25692	introduction
5	http://www.canacoll.org/	material and methods
6	http://www.humboldt.org.co/iavh/inicio	material and methods
7	http://www.mz.usp.br/	material and methods
8	http://www.leica-microsystems.com	material and methods
9	http://maps.google.com/	material and methods
10	http://goo.gl/	material and methods
11	http://goo.gl/LJ1hd	material and methods
12	http://datadryad.org/	material and methods
13	http://delta-intkey.com/	material and methods
14	http://purl.bioontology.org/ontology/HAO	material and methods
15	http://goo.gl/LJ1hd	material and methods
16	http://purl.oclc.org/NET/hymontology/proof	results
17	http://evanioidea.info/public/taxon_name/show/29154	results
18	http://www.mapress.com/zootaxa/2007f/z01496p030f.pdf	references
19	http://dx.doi.org/10.1371/journal.pone.0015991	references
20	http://purl.oclc.org/NET/hymontology	references
21	http://geolmag.geoscienceworld.org/cgi/content/extract/137/4/472-a	references
22	http://api.hymao.org/projects/32/public/ontology_class/show/5025	terminology
23	http://api.hymao.org/projects/32/public/ontology_class/show/1080	terminology
24	http://api.hymao.org/projects/32/public/ontology_class/show/3191	terminology
25	http://api.hymao.org/projects/32/public/ontology_class/show/611	terminology
26	http://hymglossary.tamu.edu/projects/32/public/ontology_class/show/1069	terminology
27	http://api.hymao.org/projects/32/public/ontology_class/show/3836	terminology
28	http://api.hymao.org/projects/32/public/ontology_class/show/4502	terminology
29	http://api.hymao.org/projects/32/public/ontology_class/show/4160	terminology
30	http://api.hymao.org/projects/32/public/ontology_class/show/1084	terminology
31	http://api.hymao.org/projects/32/public/ontology_class/show/1779	terminology
32	http://api.hymao.org/projects/32/public/ontology_class/show/5973	terminology
33	http://api.hymao.org/projects/32/public/ontology_class/show/1118	terminology
34	http://api.hymao.org/projects/32/public/ontology_class/show/1076	terminology
35	http://api.hymao.org/projects/32/public/ontology_class/show/470	terminology
36	http://api.hymao.org/projects/32/public/ontology_class/show/516	terminology
37	http://api.hymao.org/projects/32/public/ontology_class/show/4001	terminology
38	http://api.hymao.org/projects/32/public/ontology_class/show/3172	terminology
39	http://api.hymao.org/projects/32/public/ontology_class/show/522	terminology
40	http://api.hymao.org/projects/32/public/ontology_class/show/1196	terminology
41	http://api.hymao.org/projects/32/public/ontology_class/show/484	terminology
42	http://api.hymao.org/projects/32/public/ontology_class/show/526	terminology
43	http://api.hymao.org/projects/32/public/ontology_class/show/8063	terminology
44	http://api.hymao.org/projects/32/public/ontology_class/show/615	terminology
45	http://api.hymao.org/projects/32/public/ontology_class/show/1228	terminology
46	http://api.hymao.org/projects/32/public/ontology_class/show/853	terminology
47	http://api.hymao.org/projects/32/public/ontology_class/show/7261	terminology
48	http://api.hymao.org/projects/32/public/ontology_class/show/3270	terminology
49	http://api.hymao.org/projects/32/public/ontology_class/show/3241	terminology
50	http://api.hymao.org/projects/32/public/label/show_via_name/mesoscutum	terminology
51	http://api.hymao.org/projects/32/public/ontology_class/show/1711	terminology
52	http://api.hymao.org/projects/32/public/ontology_class/show/1708	terminology
53	http://api.hymao.org/projects/32/public/ontology_class/show/488	terminology
54	http://api.hymao.org/projects/32/public/ontology_class/show/3274	terminology
55	http://api.hymao.org/projects/32/public/ontology_class/show/1417	terminology
56	http://api.hymao.org/projects/32/public/ontology_class/show/1635	terminology
57	http://api.hymao.org/projects/32/public/ontology_class/show/492	terminology
58	http://api.hymao.org/projects/32/public/ontology_class/show/1075	terminology
59	http://api.hymao.org/projects/32/public/label/show_via_name/malar%20space	terminology
60	http://api.hymao.org/projects/32/public/label/show_via_name/mesosternum	terminology
61	http://api.hymao.org/projects/32/public/ontology_class/show/3173	terminology
62	http://api.hymao.org/projects/32/public/ontology_class/show/622	terminology
63	http://api.hymao.org/projects/32/public/ontology_class/show/3181	terminology
64	http://api.hymao.org/projects/32/public/ontology_class/show/532	terminology
65	http://api.hymao.org/projects/32/public/label/show_via_name/metapleuron	terminology
66	http://api.hymao.org/projects/32/public/ontology_class/show/3332	terminology
67	http://api.hymao.org/projects/32/public/ontology_class/show/623	terminology
68	http://api.hymao.org/projects/32/public/ontology_class/show/1698	terminology
69	http://api.hymao.org/projects/32/public/ontology_class/show/601	terminology
70	http://api.hymao.org/projects/32/public/ontology_class/show/3255	terminology
71	http://api.hymao.org/projects/32/public/ontology_class/show/541	terminology
71	http://api.hymao.org/projects/32/public/ontology_class/show/1597	terminology
73	http://api.hymao.org/projects/32/public/ontology_class/show/3232	terminology
74	http://api.hymao.org/projects/32/public/ontology_class/show/1780	terminology
75	http://api.hymao.org/projects/32/public/label/show_via_name/propodeum	terminology
76	http://api.hymao.org/projects/32/public/ontology_class/show/1699	terminology
77	http://api.hymao.org/projects/32/public/ontology_class/show/3174	terminology
78	http://api.hymao.org/projects/32/public/ontology_class/show/489	terminology
79	http://api.hymao.org/projects/32/public/ontology_class/show/4538	terminology
80	http://api.hymao.org/projects/32/public/ontology_class/show/3258	terminology
81	http://api.hymao.org/projects/32/public/ontology_class/show/3183	terminology
82	http://api.hymao.org/projects/32/public/ontology_class/show/4542	terminology
83	http://api.hymao.org/projects/32/public/ontology_class/show/860	terminology
84	http://api.hymao.org/projects/32/public/ontology_class/show/4591	terminology
85	http://api.hymao.org/projects/32/public/ontology_class/show/550	terminology
86	http://api.hymao.org/projects/32/public/label/show_via_name/speculum	terminology
87	http://api.hymao.org/projects/32/public/label/show_via_name/scutoscutellar%20sulcus	terminology
88	http://api.hymao.org/projects/32/public/ontology_class/show/583	terminology
89	http://api.hymao.org/projects/32/public/ontology_class/show/580	terminology
90	http://api.hymao.org/projects/32/public/ontology_class/show/4382	terminology
91	http://api.hymao.org/projects/32/public/ontology_class/show/3458	terminology
92	http://api.hymao.org/projects/32/public/ontology_class/show/610	terminology
93	http://api.hymao.org/projects/32/public/ontology_class/show/612	terminology
94	http://api.hymao.org/projects/32/public/ontology_class/show/579	terminology
95	http://api.hymao.org/projects/32/public/label/show_via_name/articulation	terminology
96	http://api.hymao.org/projects/32/public/ontology_class/show/655	terminology
97	http://api.hymao.org/projects/32/public/ontology_class/show/7242	terminology
98	http://api.hymao.org/projects/32/public/ontology_class/show/608	terminology
